# How oligoclonal are germinal centers? A new method for estimating clonal diversity from immunohistological sections

**DOI:** 10.1186/1471-2105-14-S6-S8

**Published:** 2013-04-17

**Authors:** Jose Faro, Michal Or-Guil

**Affiliations:** 1 Department of Biochemistry, Genetics and Immunology, University of Vigo, Vigo, 36310, Spain; 2 Estudos Avançados de Oeiras, Instituto Gulbenkian de Ciência, Oeiras, 2781-901, Portugal; 3 Systems Immunology Lab, Department of Biology, Humboldt-Universität zu Berlin, 10115 Berlin, Germany; 4 Research Center ImmunoSciences, Charité - Universitätsmedizin Berlin, 10115 Berlin, Germany

## Abstract

**Background:**

The germinal center (GC) reaction leads to antibody affinity maturation and generation of memory B cells, but its underlying mechanisms are poorly understood. To assemble this puzzle, several key pieces of information are needed, one in particular being the number of participating B cell clones. Since this clonal diversity cannot be observed directly, earlier studies resorted to interpreting two types of available experimental data: Immunohistology of GCs containing two phenotypically distinct B-cell populations, and antibody gene sequences of small B-cell samples from GCs. Based on a simple model, investigators concluded that a typical GC was seeded by 2-8 B cells, endorsing the current notion that GCs are oligoclonal from the onset.

**Results:**

A re-evaluation of these data showed that the used simple model is not statistically consistent with the original data. From an analysis of the experimental system, we propose a new model for estimating GC clonal diversity, including the initially neglected sampling and measurement errors, and making more general assumptions. Consistency analysis with the new model yielded an estimation of sampling and measurement errors in the experimental data of 10-11% for one B-cell population and 62-64% for the other population, and an average number of 19-23 seeder B cells. An independent analysis of antibody gene sequences of small B-cell samples from GCs, using an adapted Yule estimator of diversity, yielded a minimum estimation of 20-30 GC founder B cells, confirming the previous results.

**Conclusions:**

Our new experimental-based model provides a highly improved method to estimate the clonal diversity of GCs from inmunohistochemistry data of chimeric animals. Calculations based on this model, and validated by an independent approach, indicate that GCs most likely contain broadly varying numbers of different B cell clones, averaging 5- to 10-fold more clones than previously estimated. These findings, in line with recent results showing that GC sizes and life times are also subject to high variability, dramatically change the picture of GC dynamics.

## Background

Higher vertebrates have evolved a complex immune system that is instrumental in their protection from toxic and infectious entities. To cope with those entities a great diversity of B cell receptors is generated by the immune system. During immune responses to protein-containing antigens (Ags) a set of processes is triggered that further increases the initial Ag-specific B-cell repertoire, where the major mechanism involved is somatic hypermutation (SHM) of antibody (Ab) encoding genes [1]. These processes take place in dynamic, transient anatomical structures, so-called germinal centers (GCs). GCs are generated within primary follicles of secondary lymphoid organs during humoral immune responses [2,3].

Immunization with a protein-containing Ag induces accumulation of the Ag onto so-called follicular dendritic cells in B-cell zones (primary follicles) [4-6]. This occurs via active transport of Ag complexed to antibodies and complement factors. During this initial phase, Ag-specific T and B cells are induced to migrate and meet at the border betwen B-cell and T-cell zone [7,8]. After a period of B-T cell interaction and proliferation some Ag-specific B cells migrate back toward the center of a follicle [7,8]. Each of those B cells originate a clone, that is, a progeny of B cells with the same rearranged immunoglobulin (Ig) heavy (*Igh*) and light (*Igl*) variable (V)-region sequences. This initiates the formation of GCs, which are characterized in the first half of the GC reaction by intense B cell proliferation and increasing apoptosis [9]. Currently, it is established that GCs are the major site of memory B cell generation and SHM [2,3,10]. In addition, accumulated data over the last 15 years demonstrate that an inter- and intraclonal competition between Ag-specific B cells, whether Ab-mutated or not, takes place in GCs, accounting for a process that increases Ab affinity for Ag [11-13].

Determining the B cell clonal diversity of GCs has long been considered a starting point for the investigation of GC selection mechanisms. For that end, two experimental approaches have been classically followed. The first approach, an ingenious strategy, was to estimate the clonal diversity indirectly by measuring only a qualitative GC trait. A second approach directly measured *Igh *and/or *Igl *V-region sequence diversity in individual GCs. The conclusion drawn from the analysis of those experiments is that GCs are founded oligoclonally by about 2-8 precursor cells.

### The indirect experimental approach and the basic model

The indirect approach is based on the assumptions that every GC is founded by *n *B cells, and that the founder B cells expand at equal rates. Under these assumptions, the number of founder B cells *n *is estimated and taken as an upper limit for the number of clones.

This method is applicable in animal models that possess phenotypically distinguishable B cell subpopulations. These subpopulations should bear a similar clonal diversity and a similar propensity to be selected by Ag contact.

**Figure 1:**
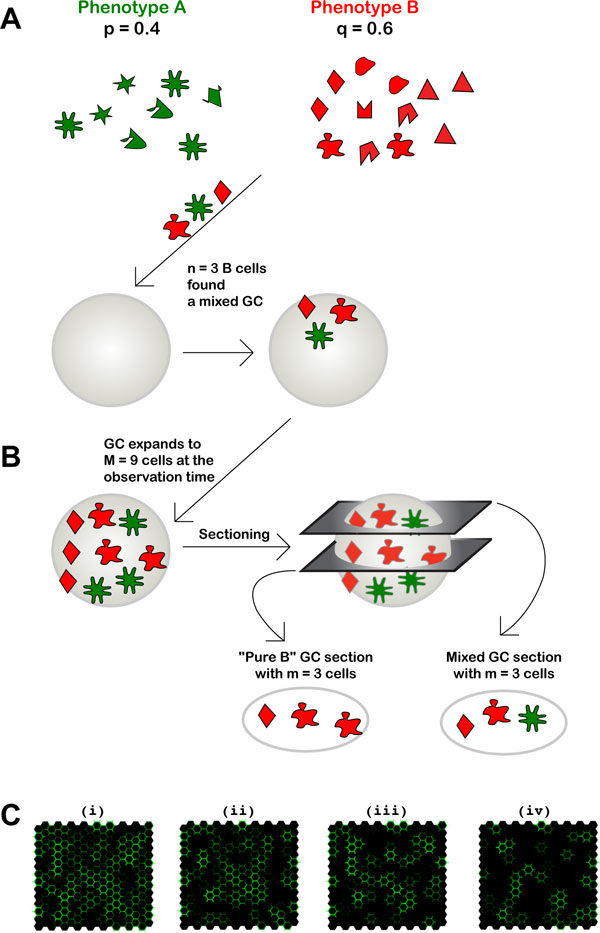
**Illustration of model parameters and immunohistological interpretation of sections from mixed GCs**. A, Cartoon showing B cells from a mixed population with phenotypes A and B founding a GC. B, Cartoon representing B cell expansion in a mixed GC, and the possible appearance of its immunohistological sections. C, Simulation of immunofluorescence staining of GC sections with Abs for B cells of type A allotype. Each panel simulates a GC section with a total of 202 cells, of which 80% are assumed to be randomly localized B lymphocytes [8,20]. Non-homogeneous staining of cells, as is typically observed, is also incorporated. Panels (i) to (iv) are, respectively, from GCs with 100%, 70%, 50%, and 40% B cells of type A.

In the simplest case, the B cells consist of two phenotypically distinguishable subpopulations, *A *and *B*. The basic model that was used is the following: Let us call *q *and *p *=1 − *q *the fractions of subpopulations *A *and *B *within total B cells, respectively. The values *q *and *p *indicate thus the prevalence with which GCs are seeded by one or the other subpopulation (see Figure 1A). Since GCs were assumed to be seeded by *n *B cells, the expected fraction of GCs seeded by *k *cells of type *A *and *n *− *k *cells of type *B *is:

(1) $$\left(\begin{array}{c} n\\ k \end{array}\right)p^{n-k}\;q^{k}.$$

Thus, the fractions of GCs expected to be seeded by a mixture of *A *and *B *cells (*f_Mix_*), only *A *cells (*f_A_*) or only *B *cells (*f*_*B*_) are, respectively,

(2) $$f_{A}\;\;=\;\;q^{n}$$

(3) $$f_{B}\;\;=\;\;p^{n}$$

(4) $$f_{Mix}=1-q^{n}-p^{n}.$$

Using either one of those three equations one can calculate *n *for given observed values of *f_A_*, *f_B _*or *f_Mix _*(denoted here as $f_{A}^{ob}$, $f_{B}^{ob}$ and $f_{Mix}^{ob}$) and known fractions *p *and *q*. Researchers developed two different experimental systems to allow for identification of two B cell subpopulations and, consequently, quantification of *p*, $f_{A}^{ob}$, $f_{B}^{ob}$ and $f_{Mix}^{ob}$:

*i) Immunization with two Ags*. In this experimental approach, two different Ags are injected together and the ensuing GC reactions are analyzed [7,14]. In one experimental setting [14], rats were immunized with spider crab hemocyanin and 4 weeks later were challenged with a mix of dinitrophenyl (DNP) coupled to spider crab hemocyanin and 2-phenyloxazolone (Ox) linked to another batch of the same protein. Three days later, spleen sections were prepared for immunohistology and the numbers of GCs specific only for Ox or DNP were counted. In a variant setting [7], (4-hydroxy-3-nitrophenyl)acetyl (NP) coupled to chicken gamma globulin (CG) was injected into (C57BL/6×BALB/c)F_1 _mice (*Igh*^b/a^). In this setting, most B cells specific for NP are expected to express λ_1 _L chains associated to *Igh*^b ^. Subsequently, it was quantified how many GCs displayed B cells that bind CG and express *λ*_1_, or either bind CG or express *λ*_1_.

These experimental systems share important problems such as the unknown but expected differences in initial numbers of Ag-specic B cells, their respective Ag affinities, and the differing valence of the Ags (in the first system, Ox *vs *DNP, and in the second system, NP *vs *CG epitopes). These are important unknowns that may greatly affect the estimation of the pre-immune fractions p and q of Ag-specific B cells, and the differential response quality of reacting Ag-specific B cells before the initiation of the GC reaction.

*ii) Two sets of B cells with distinct phenotype*. Historically this was the first strategy, and the seminal papers of Kroese and colleagues are the most cited [15-17]. In those works, rats with a mixture of two lymphocyte populations bearing different alleles of a given membrane protein were used. The authors implemented this strategy in two different system variants. In the earlier one, rats were lethally X-irradiated and reconstituted with different ratios of thoracic duct lymphocytes from both allotypes, then immunized with sheep erythrocytes, and 5 to 10 days later their spleens were analyzed for GCs [15,16]. An important source of error in this system is that working with cells *ex vivo *and injecting them at given proportions into X-irradiated hosts leaves the actual numbers of functional B cells highly uncertain. Therefore, the authors later developed a rat hemopoietic chimera model [17]. Here, embryonic liver cells from PVG RT7.2 rats were transferred into congenic PVG (RT7.1) newborn rats. Similarly to the earlier reports, GCs of immunized chimeric rats were analyzed for only presence/absence of RT7.2 cells. A main advantage of the rat hemopoietic chimeric model is that it uses normal animals, without *ex vivo *cell manipulation, because the fractions of B cells carrying each allele can be determined accurately. Therefore, this work and data are the best from a quantification perspective, and hence we will focus exclusively on it. The best set of data from this chimeric approach comes from an animal in which the ratio of donor to host cells was 21: 79 (*i.e., p *= 0.21 and *q *= 0.79). In this animal, GC sections at day 5 after immunization were found to be either pure for population A (allotype RT7.1), pure for population B (allotype RT7.2), or with a mixed population, with fractions, respectively, of$f_{A}^{ob}=0.14$, $f_{B}^{ob}=0.06$, and $f_{Mix}^{ob}=0.80$. Using this experimental data and the basic model, the authors estimated the number of founder cells as *n_A _*≈ 8 (using Eq. (2)) and *n_B _*≈ 2 (using Eq. (3)).

In that study, however, there are several potential sources of error. Firstly, measurement errors relative to the estimation of pure or mixed GCs may occur. Secondly, since data only comprise a small sample of whole GCs, effects of sampling have to be considered. Thirdly, if the conclusion stems from indirect experimental observation, then the validity of the assumptions must be assessed. Finally, an average description of many events can be erroneously taken to reflect the behavior of individual events [18].

In this paper, we will analyse those sources of error, as was motivated in [19], and that can be large enough to give misleading results. Moreover, we present a new method for estimating the clonal diversity of GC founder B cells from immunohistological sections, taking into account for the first time stochastic sampling effects and measurement errors in those apparently simple quantitative experimental designs as well as the possibility of large variability between individual GCs.

## Results

### Consistency test for the indirect experimental data and a binomial model

We first assess the question if the experimental findings in [17] are consistent with the basic model used there. The experimental data there contains more information than was used to calculate *n*. Therefore, we ask if the proportions of pure and mixed type GCs estimated in [17] do indeed correspond to a binomial distribution. To answer that, we compare in Table 1 the experimental data in [17] with the corresponding expected quantities obtained utilizing either the estimated *n_A _*or *n_B _*and Eqs. (2-4). There, the respective χ^2 ^values are also shown. A χ^2 ^equal to 10.827 corresponds to a probability of 0.001 for 1 df. However, the resulting χ^2 ^are considerably greater than that value, which shows how inconsistent model and reported values are. This reveals the need for a more detailed, new model, which should take into account sampling and measurement errors, and relax the assumption that all GCs are founded by the same number *n *of B cells.

**Table 1:** Analysis of internal consistency of data in [17] used to estimate *n *from chimera with *p *= 0.21. χ^2 ^of observed *vs *expected fractions *f*_*A*_, *f*_*B *_and *f_Mix _*were calculated taking either observed $f_{A}\;\left(f_{A}^{ob}\right)$ or observed $f_{B}\;\left(f_{B}^{ob}\right)$ as the reference values.

	$n_{A}={\text{log}}_{\left(1-p\right)}\left(f_{A}^{ob}\right)$				$n_{B}={\text{log}}_{p}\left(f_{B}^{ob}\right)$		
			
		**(%)**				**(%)**	
	Observed		Expected*^a^*		Observed		Expected*^b^*
*f_A_*	14		-		14		65.4
*f_B_*	6		2:2 × 10^−4^		6		-
*f_Mix_*	80		86.0		80		28.6
*X*^2^		161996*^c^*				132.6*^c^*	

*^a^*Expected *f_B _*was calculated as $p^{n_{A}}$.

*^b^*Expected *f_A _*was calculated as ${\left(1-p\right)}^{n_{B}}$.

*^c^*χ^2^(prob.: 0.001; df: 1) = 10.827.

### New model for indirect, immunohistological data

**Figure 2:**
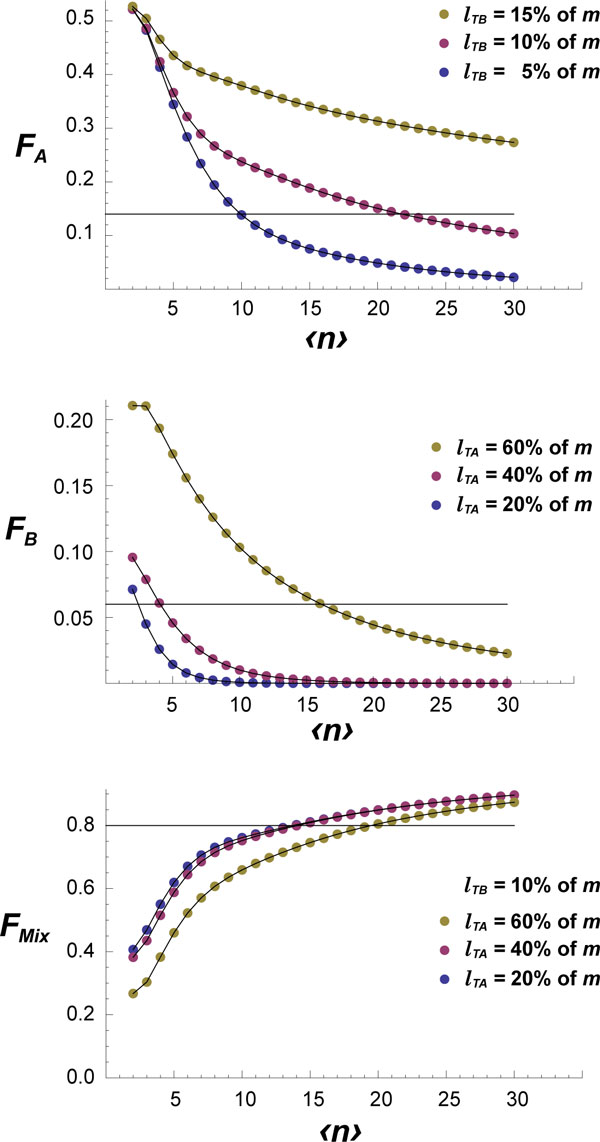
**Theoretical estimations of the expected fractions of type A, type B and mixed GC sections**. Fractions of GC sections scored as type *A *(*F_A_*), type *B *(*F_B_*) or mixed (*F_Mix_*), were estimated using the new model (Eqns. 5-12) for different values of scoring errors $l_{T_{A}}$ and $l_{T_{B}}$. 〈*n*〉, average number of GC founding B cells; $l_{T_{A}}$, scoring error for B cells of type *B*, and $l_{T_{B}}$, scoring error for B cells of type *A*. Calculations were performed assuming a GC size of M = 5000 cells, and *m *= 100 cells in a section. Horizontal lines correspond to the observed fractions in [17].

The new, immunohistological trait-based, model was used to estimate the fractions of GC sections expected to be scored as type *A *(*F_A_*), type *B *(*F_B_*) or mixed (*F_Mix_*), for different values of the average number 〈*n*〉 of GC founder B cells. Thus, a systematic analysis of Eqs. 12 was performed for varying values of *M*, the size *m *of GC sections, and scoring errors, $l_{T_{A}}$ and $l_{T_{B}}$. A representative example of the obtained results is shown in Figure 2 for *M *= 5000 cells and *m *= 100 cells. There, the upper, middle and bottom panels correspond, respectively, to the fractions *F_A_*, *F_B _*and *F_Mix_*. In each panel three dotted curves are shown corresponding to the three values indicated there for $l_{T_{B}}$ or $l_{T_{A}}$.

We then asked if the data on pure and mixed GC sections in [17] are consistent under the new model. A χ^2 ^test was used again to compare the values reported in the original paper with the expected values obtained with the new model as defined by Eqns. 5-12. For $l_{T_{A}}=l_{T_{B}}$, in all cases the χ^2 ^test was equal or higher than 33.452 (χ^2 ^= 13.815, for *p *= 0.001 and *df *= 2), indicating that the reported experimental values are too different from the expected ones to be due to random variations. These differences can be due to the fact that the detecting methods for each of the two populations are different, suggesting that the respective scoring errors are also different. As previously mentioned, T cells account for 10-20% of GC cells [8,20]. This indicates that the scoring error for B lymphocytes of type A must be $l_{T_{B}}\geq 10\%$ of *m*. Assuming the minimum value for $l_{T_{B}}$, that is, $l_{T_{B}}=10\%$ of *m*, and *m *= 100 cells/section, the value of 〈*n*〉 for which $F_{A}=F_{A}^{ob}\left(=0.14\right)$ is: 〈*n*〉 = 21-22 (Figure 2, upper panel, cross of magenta dotted curve and black horizontal line). Moreover, the value of 〈*n*〉 increases with the scoring error $l_{T_{B}}$.

On the other hand, the results shown in Figure 2, middle panel, suggest that for 〈*n*〉 = 21—22 cells the scoring error for B lymphocytes of type B, $l_{T_{A}}$, must be higher than 40% of *m *so that the expected fraction *F*_*B *_is not too far from the observed value $F_{B}^{ob}=0.14$. Indeed, a systematic calculation of the χ^2 ^for *m *= 200 cells and discrete values of $l_{T_{B}}$ from 10 to 15%, and $l_{T_{A}}$ from 50 to 65% showed several local minima. The two main χ^2 ^minima were: χ^2 ^= 0.027, for 〈*n*〉 = 19, $l_{T_{B}}=10\%$, $l_{T_{A}}=62\%$ and χ^2 ^= 0.0048, for 〈*n*〉 = 23, $l_{T_{B}}=11\%$, $l_{T_{A}}=64\%$. These χ^2 ^values are well below the χ^2 ^for *p *= 0.001 and 2 df (= 13.815). Thus the predicted *average *number of GC founder B cells given by this new model is 〈*n*〉 = 19 − 23 cells. Note that the actual initial clonal diversity of a large fraction of GCs can be considerably higher than those average values, as that diversity is assumed to obey a Poisson distribution.

### Validation of the new model by direct experimental evidence

B cell clonal diversity in individual GCs can be directly determined by analyzing rearranged *V *-gene sequences from either *Igh *or *Igl *loci. Such an investigation aims at isolating small B cell aggregates from individual GC sections and sequencing *V *-gene segments from defined *V *families.

In the literature, the most cited data relates to sequences of rearranged CDR3s from the *Igh *locus obtained at day 16 or earlier after primary immunization of C57BL/6 mice against NP coupled to protein carriers [12,21]. Here, the analysis of individual GCs comprised the following tasks: a) from each analyzed GC section take a cell aggregate (sample *A*) of ~50 cells; b) choose a *V_H _*gene family and amplify related sequences by PCR; c) take a very small sample (sample *B*) out of the resulting PCR products (usually 10^−10^%-10^−8^% of all sequences); d) sequence ≈ 100% of sample *B*. Between 1 and 9 different sequences were found in each GC section, corresponding to 1-9 different clones. These numbers are often cited as reflecting the clonal diversity of an average GC.

However, statistical methods can be used to obtain a better estimation of the number of clones present in sample *A *[19]. The data can then be analyzed with statistical approaches like the abundant coverage estimator (ACE) [22], or the Yule-Simon method [23,24].

These techniques, borrowed from ecology, have already been used to estimate the CDR3 diversity of naive CD4^+ ^T cell subpopulations [25,26]. It is important to know, however, that they tipically underestimate true diversity, especially ACE, which often results in quite low estimates [26]. We therefore applied the Yule-Simon estimator to different experimental data on GC derived *V *-gene sequences. The resulting estimations of GC B cell diversity is shown in Tables 2 and 3. They suggest that after day 10 of a primary IR there is a severe reduction of diversity (Table 3). Importantly, this is a time when selection is thought to begin to operate, reducing and biasing GC clonal structure [27]. However, the Yule-Simon method estimated a diversity before the start of that reduction of 20-30 clones (Table 3).

**Table 2:** Clonal diversity estimation of B cells in GC sections using the Yule method [26].

	Diversity	
	
GC	Observed	Estimated
B8	5	19
B12	4	13
B15	2	3

GCs are from day 10 of a primary IR. Data from [21].

**Table 3:** Clonal diversity estimation of B cells in GC sections using the Yule method [26].

		Diversity	
		
day	GC	Observed	Estimated
4	H4	3	7
6	J5	9	29
8	M16	1	18
10	B8	6	32
14	D2	2	3
16	61AA02	2	4

GCs are from different days of a primary IR. Data from [12].

Notice also that such diversity corresponds to GC sections, themselves small portions of GCs. Consequently, it can be reasonably expected that the obtained diversity estimations are significantly smaller than the diversity in whole GCs. In addition, since the experimental analysis focused on particular *VJ *rearrangements, polyclonality was even further underestimated. Thus, contrary to the classical interpretation that the experimental data supported the oligoclonal concept of GC seeding, a more rigorous statistical analysis points to a GC clonal diversity before contraction of about 10-fold greater than the usually cited values of 2-8 clones.

## Discussion

Here, we re-assess the classical methodology of estimating GC clonal diversity. Earlier reports estimated the number of different GC B cell clones by determining the number of founder B cells as its upper limit. In those works, the number of GC founder B cells was indirectly determined using immunohistology, together with a simple model which assumed a Bionomial distribution for the proportions of GCs founded by either a mixture or only one of two B cell subpopulations (that could be distinguished phenotypically). Key assumptions of this method are that the effects of proliferation and selection are similar in both populations, and that Ag-specific B cells do not enter continuously into GCs. Based on that model, it was concluded that GCs are seeded by 2-8 B cells, and hence are oligoclonal [17].

Re-assessing the whole approach led us to a more general mathematical model that takes into account: a) sampling errors, b) scoring errors in classification of GC sections, and c) variable seeding B cell numbers. By fitting this extended model to a Poisson seeder cell distribution, we obtained a mean and variance of 19-23 cells. Also, our analysis indicates very different values for the scoring errors $l_{T_{A}}$ and $l_{T_{B}}$. More specifically, $l_{T_{A}}=10-11\%$ and $l_{T_{B}}=62-64\%$. A χ^2^-test showed that the experimental data and the extended model are consistent under these conditions. The range of GC seeder B cell numbers also matches very well the range resulting from the statistical analysis of different *V *-gene sequences determined directly. In earlier reports, 1-9 different clones were inferred from *V *-sequences present in sections of murine GCs [12,21]. However, a diversity analysis based on the Yule-Simon method indicates that the observed *V *-gene sequence diversity corresponds to more than 20 different B cell clones in individual GC sections, which in turn comprise a small fraction of the whole GC volume.

These results are supported by other observations. It has been previously reported that GC seeder B cells include cells with very low affinity for Ag, which can be outcompeted by higher affinity B cells [28,29], making likely that they are missed in small B cell samples of GCs. Moreover, an analysis of the GC B cell diversity in chickens, during a primary immune response, concluded that more than 20 B cells seeded the observed GCs [30]. Finally, computer simulations of B-cell affinity maturation also indicated that GCs are founded by more than 30 B cells [31].

## Conclusion

We conclude that the ensemble of GCs is subject to a high variation in clone numbers, the average GC clonal diversity being around 10-fold higher than previously thought, and hence that oligoclonality is not a generic trait of GCs.

It can be argued that the present model is based on some assumptions that may be too rigid. For instance, for the sake of simplicity, GCs were assumed here to contain 5000 B cells, but we know that the variation in GC sizes is very large, ranging from small GCs containing less than 100 to a few GCs containing up to 20,000 B cells [32,33]. Including this additional distribution in the analysis would render the predicted clonal diversity even broader.

Another possible refinement in the model concerns the assumption that GCs are closed entities. Indeed, recently, theoretical studies by Or-Guil and colleagues [18], as well as in vivo imaging studies by Nussenzweig and colleagues [34] and Haberman and colleagues [35] suggest that GCs are open structures, receiving frequent visits from follicular B cells, and capable of recruiting high affinity Ag-specific B cells. This is in agreement with another recent study of GC samples microdissected from three reactive human lymph nodes [36]. In this study, analysis of IgM and IgG *V_H _*transcripts detected single hypermutated B cell clones in multiple GCs, implying B cell migration between GCs. A further study shows that when high affinity cells are deleted from mature GCs, a diverse lower affinity subpopulation re-emerges [37], indicating that GCs are either polyclonal, or open, or both. Taking continuous B cell entry into account in the present model for indirect observation would mean that different clone sizes have to be included. The present model represents then only an estimation based on the large clone sizes easily observable by their phenotypic traits, giving us the lower limit for clonal diversity.

All this reveals an yet to be explored link between the dynamics of GC clonal diversity and B cell migration and selection in GCs. Thus, it can be expected that gaining deeper insights into clonal diversity, its distribution and dynamics will help to shed more light into key processes involved in GC selection.

## Methods

### Implementation of a new model for estimating the expected fractions of single and mixed GCs from the indirect immunohistochemistry approach

The classical reports of the indirect immunohistochemistry approach mentioned above ([15-17]) took sections to represent whole GCs. However, sections are small samples of GCs, and as such do not always reflect the true GC composition. Consider, for instance, a GC seeded by *k *cells of type *B *and *n *− *k *cells of type *A*, and totaling *M *cells. This GC will have $M(1-\frac{k}{n})$ cells of type *A*. However, a section containing *m *cells $(\text{with }m\leq M(1-\frac{k}{n})$ will consist, with a probability $\left(\begin{array}{c} M\left(1-\frac{k}{n}\right)\\ m \end{array}\right)/\left(\begin{array}{c} M\\ m \end{array}\right))$, exclusively of type *A *cells, feigning a single population GC (see Figure 1B). This shows that sampling errors lead to overestimating the number of single population GCs.

Errors of observation can lead to a further overestimation. For instance, T cells account for 10-20% of GC cells [8,20]. Therefore, experimenters in ref. [17] set a subjective and arbitrary cut-off for counting mixed GCs due to T cells possibly being counted as B cells. That is, GC sections with a minimal number of donor type cells were counted as "pure" host type [17]. Finally, if only one population is analyzed for its presence, and the other B cell population is simply inferred, as in [15-17], it can be that a GC section is scored as 100% positive for the former population because all cells are apparently labeled, while in fact it contains cells of the latter population. Actual absence of the second population can only be determined by staining for that population.

This problem is illustrated in Figure 1C. In a computer simulation of immunofluorescence staining of GC sections, B cells sampled from a two-phenotype population, *A *and *B*, were randomly placed on a hexagonal lattice representing a GC section. To account for GC T cells and other non-B cells, only 80% of the lattice was filled with B cells. Following the immunofluorescence staining procedure of Hermans *et al *[17], only the B cell phenotype *A *was visualized (green color). Different fractions of cells of type *B *illustrate how subjective the classification of GCs as not containing B lymphocytes of type *B *is. Note that panels I-III, representing GCs with 100%, 70%, 50%, type *A *B cells, look quite similar. Only in panel IV, representing a GC section with 40% B cells of type *A*, do lower numbers of type a *A *cells become evident, indicating the presence of cells of type *B*. (All simulations and calculations described here were performed with the program Mathematica^®^.)

To take such measurement errors into account, we introduced a parameter *l_T _*≤ *m *representing the maximum number of cells that can be overlooked. In other words, sections with up to *l_T _*B cells of one type are scored as pure sections of the other type. Hence, to account for all sections that look pure, we have to sum over all probabilities of finding mixed sections with *l_T _*or less B cells.

Taking into consideration both sampling and measurement errors described above, we extended Eq. (1) according to the ansatz described in [19]. We found that the probability to find a mixed GC, seeded by *k *cells of type B (1 ≤ *k *<*n*), yielding an apparently pure section of type *A *is:

(5) $$P_{A}\left(n,k\right)=\left(\begin{array}{c} n\\ k \end{array}\right)p^{k}\;q^{n-k}\overset{min\left[l_{T_{B}},r\right]}{\underset{l=max\left[0,m-s\right]}{\sum }}\frac{\left(\begin{array}{c} s\\ m-l \end{array}\right)\;\left(\begin{array}{c} r\\ l \end{array}\right)}{\left(\begin{array}{c} M\\ m \end{array}\right)}$$

where $r=\frac{k}{n}M$ and *s *= *M *− *r *are, respectively, the number of cells of type *B *and type *A *in that GC, and *m *is the total number of B cells in that section.

Accordingly, the probability to find a mixed GC seeded by *k *cells of type *A *but yielding an apparently pure section of type *B *is:

(6) $$P_{B}\left(n,k\right)=\left(\begin{array}{c} n\\ k \end{array}\right)p^{k}\;q^{n-k}\overset{min\left[l_{T_{A}},s\right]}{\underset{l=max\left[0,m-r\right]}{\sum }}\frac{\left(\begin{array}{c} s\\ l \end{array}\right)\;\left(\begin{array}{c} s\\ m-l \end{array}\right)}{\left(\begin{array}{c} M\\ m \end{array}\right)}$$

Hence, the total fractions of sections expected to be scored as pure *A*, pure *B *and mixed are, respectively:

(7) $$f_{A}\left(n\right)=\overset{n-1}{\underset{k=0}{\sum }}P_{A}\left(n,k\right)$$

(8) $$f_{B}\left(n\right)=\overset{n}{\underset{k=1}{\sum }}P_{B}\left(n,k\right)$$

(9) $$f_{Mix}\left(n\right)=1-f_{A}\left(n\right)-f_{B}\left(n\right)$$

A further extension of the basic model addresses its assumption that all GCs are seeded by the same number *n *of cells. If Ag-specific B cells randomly enter nascent GCs and these events are independent of each other, then B cell seeding of GCs is better described by a Poisson distribution of clones rather than by a fixed number of clones *n *supposedly valid for all GCs. We therefore assume that the clonal diversity obeys a Poisson distribution with mean *λ *= 〈*n*〉, such that the fraction of GCs seeded by *n *B cells is:

(10) $$P\left(\lambda ,n\right)=\frac{\lambda^{n}}{n!}e^{-\lambda }$$

Then the fractions of sections expected to be scored as pure *A*, pure *B *and mixed from GCs seeded by *n *B cells are, respectively:

(11) $$\begin{array}{llll} h_{A}\left(\lambda ,n\right) & =P\left(\lambda ,n\right)\times f_{A}\left(n\right)\; & & \;\\ h_{B}\left(\lambda ,n\right) & =P\left(\lambda ,n\right)\times f_{B}\left(n\right)\; & & \;\\ h_{Mix}\left(\lambda ,n\right) & =P\left(\lambda ,n\right)\times f_{Mix}\left(n\right)\; & & \;\\ & \; & \end{array}$$

Finally, considering that *λ *= 〈*n*〉, the *total *fractions of sections expected to be scored as pure *A*, pure *B *and mixed are given by the equations:

(12) $$\begin{array}{llll} F_{A}\left(\langle n\rangle \right)\;\; & =\;\;\overset{\infty }{\underset{n=1}{\sum }}h_{A}\left(\langle n\rangle ,n\right)\; & & \;\\ F_{B}\left(\langle n\rangle \right)\;\; & =\;\;\overset{\infty }{\underset{n=1}{\sum }}h_{B}\left(\langle n\rangle ,n\right)\; & & \;\\ F_{Mix}\left(\langle n\rangle \right)\;\; & =\;\;\overset{\infty }{\underset{n=1}{\sum }}h_{Mix}\left(\langle n\rangle ,n\right)\; & & \;\\ & \; & \end{array}$$

## List of abbreviations used

GC: germinal center; Ag: antigen; SHM: somatic hypermutation; Ab: antibody; Ig: immunoglobulin; Igh: immunoglobulin heavy chain; Igl: immunoglobulin light chain; DNP: dinitrophenyl; Ox: 2-phenyloxazolone; NP: (4-hydroxy-3-nitrophenyl)acetyl; CG: chicken gamma globulin; V: variable.

## Competing interests

The authors declare that they have no competing interests.

## Authors' contributions

All authors contributed equally to this work.

## Declarations

This article has been published as part of *BMC Bioinformatics *Volume 14 Supplement 6, 2013: Selected articles from the 10th International Conference on Artificial Immune Systems (ICARIS). The full contents of the supplement are available online at http://www.biomedcentral.com/bmcbioinformatics/supplements/14/S6.
